# Visual Assessment in Elderly Inpatients Following a Fall

**DOI:** 10.7759/cureus.92473

**Published:** 2025-09-16

**Authors:** Saeed Azizi, Omar Hawwa, Ali Istanaksai, Hadyn Kankam, Nadeem Ali

**Affiliations:** 1 Ophthalmology, Moorfields Eye Hospital NHS Foundation Trust, London, GBR; 2 Medicine, St George’s University Hospitals NHS Foundation Trust, London, GBR; 3 Medicine, North Middlesex University Hospital, London, GBR; 4 Ophthalmology, London Squint Clinic, London, GBR

**Keywords:** elderly, falls, quality improvement, visual assessment, visual impairment

## Abstract

Introduction

Falls are a major cause of morbidity in older adults, with visual impairment recognized as a key modifiable risk factor. National guidelines recommend vision assessment following a fall; however, adherence remains suboptimal. This quality improvement project aimed to evaluate compliance with national recommendations and implement interventions to improve visual assessment rates in elderly inpatients.

Methods

A two-cycle retrospective cross-sectional review was conducted at a tertiary hospital in London. Electronic records of inpatients aged ≥65 admitted across two time points, one year apart, were analyzed. The first cycle assessed baseline documentation of vision assessments, defined as visual acuity (VA) or confrontational fields. Interventions included staff education sessions, distribution of visual prompts, and improved access to bedside VA tools. A re-audit was performed post-intervention to evaluate the impact.

Results

A total of 337 patient records were reviewed (181 pre-intervention; 156 post-intervention). Among patients admitted after a fall (n=51 pre-intervention, n=42 post-intervention), documented VA assessment increased from 7.8% to 16.7% (p=0.21), and confrontational visual field testing increased from 5.9% to 19% (p=0.061) post-intervention. While these changes did not reach statistical significance, they demonstrate a positive trend following low-cost interventions.

Conclusion

Visual assessments remain underperformed in elderly inpatients following a fall despite national guidance. Targeted low-cost interventions, including staff education and improved access to assessment tools, can improve compliance. Continued multidisciplinary engagement, system-level changes, and regular audit cycles are necessary to embed vision assessment as a routine component of post-fall care and to reduce fall-related harm.

## Introduction

Falls among the elderly remain a pressing public health issue, contributing significantly to morbidity, mortality, and diminished quality of life. Approximately 30% of individuals aged ≥65 years experience at least one fall annually, with rates rising to nearly 50% among those aged ≥75 years. Such falls often lead to injuries, loss of independence, reduced quality of life, and hospitalizations [1]. In the United Kingdom, falls account for more than 200,000 hospital admissions annually and cost the NHS approximately £2.3 billion per year [2]. Visual impairment is recognized as a key modifiable intrinsic risk factor for falls in older adults. Age-related diminutions in visual acuity, contrast sensitivity, and peripheral field compromise obstacle detection and balance control. These deficits heighten the likelihood of trips, slips, and injurious falls [3].

Empirical evidence supports the effectiveness of vision improvement in reducing fall risk. For instance, expedited first-eye cataract surgery in elderly women led to a 34% reduction in fall rate and a decreased incidence of fractures within 12 months [4]. Moreover, even patient-reported visual difficulties under low luminance conditions, such as contrast-sensitivity-dependent issues, have been independently associated with multiple falls, suggesting the vital role of subjective and objective visual function assessments [5].

Given the link between visual deficits and falls, national guidance recognizes the importance of assessing vision as part of a multifactorial approach to falls prevention. The National Institute for Health and Care Excellence (NICE) recommends that older adults presenting after a fall should undergo a visual assessment as part of their clinical work-up following a fall [6]. Our study aimed to address this gap through a two-cycle cross-sectional audit and intervention project in a tertiary hospital in London, focusing on patients aged ≥65 admitted after a fall. By comparing documentation of visual assessments pre- and post-intervention, we sought to evaluate whether simple strategies could meaningfully improve compliance with vision assessment guidelines and support broader QI efforts in patient safety for elderly falls.

## Materials and methods

Aim and objectives

The aim of this quality improvement project was to evaluate the extent to which vision assessments are carried out in older adults presenting with falls at a tertiary hospital in London. This initiative was undertaken in line with national guidelines, including NICE Clinical Guideline 249 (NG249), which recommends multifactorial assessment of fall risk in older adults, specifically including the evaluation of visual impairment [6]. 

The primary objective was to assess compliance with national recommendations regarding vision assessment following a fall in patients aged ≥65. A secondary objective was to increase the rate of vision screening through targeted interventions. The broader goal was to ensure patients with visual impairment were referred for appropriate follow-up, reducing fall recurrence and improving patient safety.

Standards

The audit measured performance against standards outlined in NICE NG249 and guidance from the British Geriatrics Society and the Royal College of Ophthalmologists (2011) [4]. These recommend that all older adults undergoing falls assessment should be screened for visual impairment, with visual acuity measured using a Snellen chart. A visual acuity of 6/12 or worse with correction denotes impairment. Patients with identified visual deficits should undergo a comprehensive eye examination, either as an inpatient or in an outpatient setting. Local mechanisms should be in place to promote awareness and uptake of services for individuals with visual impairment. A benchmark compliance level of one hundred percent was set for vision assessment in all elderly patients admitted following a fall.

Data collection

The initial audit was conducted as a cross-sectional review across all seven general medical and senior health wards. Electronic patient records were reviewed to identify inpatients aged sixty-five and over, and notes were reviewed to identify who had presented following a fall. No exclusions were made based on comorbidities, length of stay, or cognitive status. Data on completion of visual acuity and confrontational visual field assessments were extracted and stored securely in Microsoft Excel (Microsoft Corporation, Redmond, USA) by co-authors SA & HK. 

Interventions

Following the presentation of the audit findings, a series of interventions were implemented to increase the implementation and quality of visual assessments. Stakeholders included patients, relatives, ward nurses, junior and senior doctors, physiotherapists, and occupational therapists. Interventions comprised educational sessions for clinical staff through teaching sessions, distribution of visual prompts and reminders via email, and increased accessibility of bedside vision assessment tools by printing and placing Snellen charts on the wards, as well as raising awareness of validated smartphone applications to assist in visual assessment.

Re-audit

A re-audit was performed one year after the initial audit to assess the impact of the interventions. Data collection followed the same methodology as the initial audit to ensure consistency and comparability.

## Results

A total of 337 patient records were reviewed: 181 pre-intervention and 156 post-intervention. In the pre-intervention cohort, 51 patients (28.1%) were admitted following a fall, of whom only four patients (7.8%) had a documented visual acuity (VA) assessment, and three patients (5.9%) underwent confrontational visual field (VF) testing. In the post-intervention cohort, 42 patients (26.9%) were admitted following a fall. Of these, seven patients (16.7%) received VA assessments, and eight patients (19.0%) underwent VF testing. A summary of the results is displayed in Table 1 and Table 2. 

**Table 1:** Percentage of elderly patients who were admitted with fall

Cohort	Total Records	Patients with Fall n (%)
Pre-intervention	181	51 (28.1%)
Post-intervention	156	42 (26.9%)

**Table 2:** Vision assessment compliance Fisher's test was used for statistical significance, which was defined as p<0.05.

Assessment Type	Pre-intervention (n=51 fallers) n (%)	Post-intervention (n=42 fallers) n (%)	p-value
Visual acuity (VA)	4 (7.8%)	7 (16.7%)	0.21
Visual fields (VF)	3 (5.9%)	8 (19.0%)	0.061

Statistical comparisons between pre- and post-intervention compliance rates were performed using Fisher’s Exact Test due to the small sample sizes in some categories. Although these improvements did not reach statistical significance (p<0.05) (p=0.21 for VA, p=0.061 for VF), the results suggest an upward trend in compliance following the targeted interventions. This indicates that relatively simple, low-cost measures can positively influence clinical practice, though compliance remains below the 100% target recommended by NICE NG249 and professional guidelines.

Interpretation

The intervention resulted in more than a doubling of visual acuity (VA) assessment rates, increasing from 7.8% to 16.7%, and a threefold rise in visual field (VF) testing, from 5.9% to 19.0%. Although these changes did not reach statistical significance, the consistent trend toward improvement is encouraging. These findings underscore the challenges of implementing guideline-driven care in routine practice, particularly within high-pressure acute settings.

Next steps

Planned strategies to further improve compliance include ongoing stakeholder engagement to reinforce the importance of vision assessment in falls prevention. Additionally, integrating visual acuity and visual field assessments into standardized falls documentation and proformas will help prompt clinicians at the point of care. Training will also be embedded into staff induction programs to ensure new members are familiar with NICE recommendations from the outset. Finally, the promotion of validated smartphone-based vision assessment tools during induction is planned to reduce barriers to implementation and enhance accessibility.

## Discussion

This quality improvement project demonstrated that visual assessments are underperformed and under-documented in older adults presenting with falls, despite recommendations from NICE NG249 and guidance from the British Geriatrics Society and Royal College of Ophthalmologists. Initial audit data revealed that only 7.8% of patients who had a fall underwent visual acuity assessment, and 5.9% received confrontational visual field testing. Following targeted interventions, these figures increased to 16.7% and 19%, respectively. While this represents progress, substantial gaps remain.

Visual impairment is a well-established risk factor for falls in the elderly. Untreated refractive error and conditions such as cataracts, age-related macular degeneration, and glaucoma compromise depth perception, peripheral vision, and visual acuity, all critical for safe mobility [3]. Timely identification through bedside assessments enables referral for optometric review or further ophthalmological care, potentially reducing fall recurrence and improving outcomes [4].

Barriers to consistent visual screening include limited clinician awareness, perceived time constraints, and assumptions that assessment requires specialist input. The interventions implemented included staff education, visual prompts, and accessible assessment tools, demonstrating that simple, low-cost strategies can improve compliance.

However, the majority of patients admitted following a fall still did not receive documented visual assessments. System-level changes, including dedicated visual assessment personnel (ie, optometrist) or integration into electronic proformas and mandatory staff training, may enhance adherence. Ongoing multidisciplinary engagement with ward nurses, occupational therapists, and physiotherapists is essential to embed visual assessment as routine practice. Further cycles of audit and feedback will support monitoring and sustainability of improvements.

Study limitations

This project was limited by short data collection periods and modest sample sizes across a single tertiary hospital in London, which may restrict generalizability. Data relied on documentation in patient records; assessments may have been performed but not recorded. The study did not evaluate whether patients with identified visual deficits received appropriate follow-up, limiting conclusions regarding the downstream impact.

Study strengths

Strengths include alignment with national standards, clearly defined methodology, and consistent application across audit cycles. Interventions were low-cost, scalable, and integrated into existing clinical workflows. Multidisciplinary collaboration and increased awareness of visual risk factors contribute to a culture of patient safety and quality improvement.

## Conclusions

This quality improvement project identified a significant gap in the assessment and documentation of vision in older adults admitted following a fall. Visual assessments were poorly performed at baseline, despite national recommendations highlighting their importance in falls prevention.

Targeted low-cost interventions, including staff education and improved access to assessment tools, increased compliance with vision assessments. Embedding vision screening as a routine component of post-fall care will require continued collaboration with multidisciplinary teams, system-level integration such as electronic prompts, and regular audit cycles. Sustained implementation has the potential to reduce fall-related harm through early identification and management of visual impairment.

## References

[1] Reed-Jones RJ, Solis GR, Lawson KA, Loya AM, Cude-Islas D, Berger CS (2013). Vision and falls: a multidisciplinary review of the contributions of visual impairment to falls among older adults. Maturitas.

[2] (2025). National Institute for Health and Care Excellence (NICE). Interventions for prevention of falls in hospital settings: falls: assessment and prevention in older people and people 50 and over at higher risk: evidence review G. Interventions for Prevention of Falls in Hospital Settings: Falls: Assessment and Prevention in Older People and People 50 and Over at Higher Risk: Evidence Review G.

[3] Saftari LN, Kwon OS (2018). Ageing vision and falls: a review. J Physiol Anthropol.

[4] Harwood RH, Foss AJ, Osborn F, Gregson RM, Zaman A, Masud T (2005). Falls and health status in elderly women following first eye cataract surgery: a randomised controlled trial. Br J Ophthalmol.

[5] Terheyden JH, Gerhards J, Ost RA, Wintergerst MW, Holz FG, Finger RP (2023). Patient-reported vision impairment in low luminance predicts multiple falls. BMC Geriatr.

[6] (2025). National Institute for Health and Care Excellence (NICE). Falls: assessment and prevention in older people and in people 50 and over at higher risk. NICE Guideline NG249. https://www.nice.org.uk/guidance/ng249.

